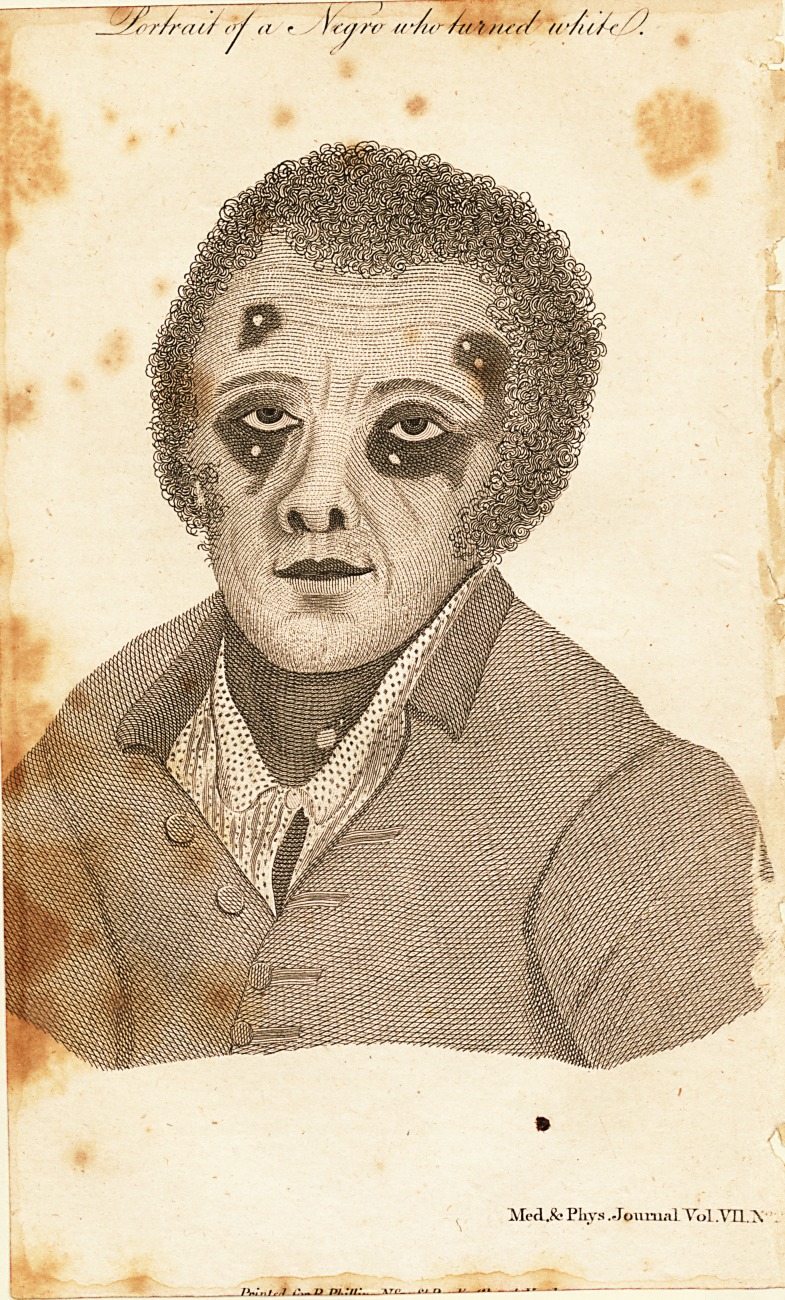# Case of a Negro Turning White

**Published:** 1802-08-01

**Authors:** T. Dancer

**Affiliations:** Kingston, Jamaica


Medical and Physical Journal.

VOL. VIII.] AUGUST 1, 1802. [NO. XLII.

### *Case of a Negro turning White; communicated by* Mr. T. DANCER, *of Kingston, Jamaica.*

[ With an Engraving. ]

IT is well known that there are Negroes, (Albinoes) born white, fome are party coloured or pie-bald, and it is not uncommon for Negroes in extreme old age to have white fpots or blotches about them; but a cafe like the following is, I believe, without example in this ifland. I have feen an account of fuch an one in America, which is perhaps the only one befides ever known or heard of.

Charles Fuller, a Negro man, between fifty and fixty years of age, belonging to Middleton eftate, in St. Thomas in the Eaft, a Creole, (that is one born in the Weft Indies) had in the month of January laft, a flight fever; in recovering from which, feveral white fpots appeared on his face, which fpreading and running into each other, his whole face now is nearly that of a white man, three or four black blotches only remaining, and his upper lip being black. The fame white fpots begin to appear on the neck, arms, and trunk; fo that, in a fhort time, the Ethiopian may become white, and the leopard change his fkin, contrary to what it is fuppofed can ever poffibly happen. No caufe whatever can be affigned for this furprizing change. The man is in perfect good health, having no fymptom of any difeafe, except a flight œdema or fwelling of the ankles, to which he for a long time has been occafionally fubject. He has been many years a hot-houfe doctor, that is, an attendant on the fick in the plantation hofpital, and has undergone no alteration in any of his habits of living. He has not been under the influence of any mental impreffion, though at prefent he is rather dejected from the circumftance of the change of colour, which he confiders as the harbinger of fome worfe alteration. The colour is a healthy ruddy white, not that of an Albinoe, nor does he labour under any defect of vifion as all the Albinoes do.

I leave it to Anatomifts and Phyfiologifts of greater abilities than myfelf, to employ their fpeculations on this curious Cafe.

## Figures and Tables

**Figure f1:**